# Supplementary material for: plantiSMASH: automated identification, annotation and expression analysis of plant biosynthetic gene clusters
Source: Nucleic Acids Res. 2017 Apr 27;45(Web Server issue):W55–63. doi: 10.1093/nar/gkx305 (PMC5570173; doi:10.1093/nar/gkx305)
Supplement: Supplementary Data [file gkx305_Supp.zip › nar-00493-web-b-2017-File006.docx]

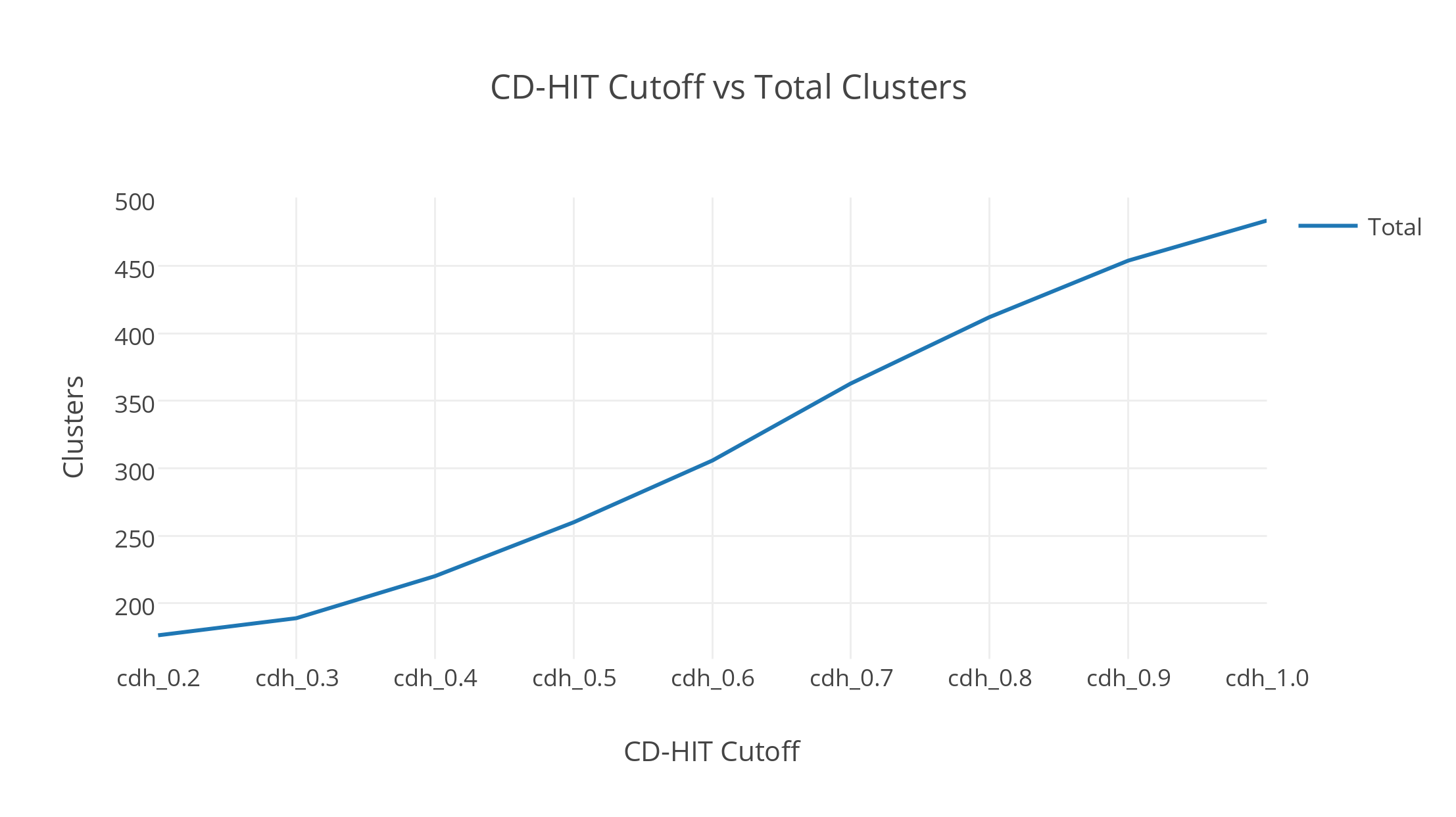

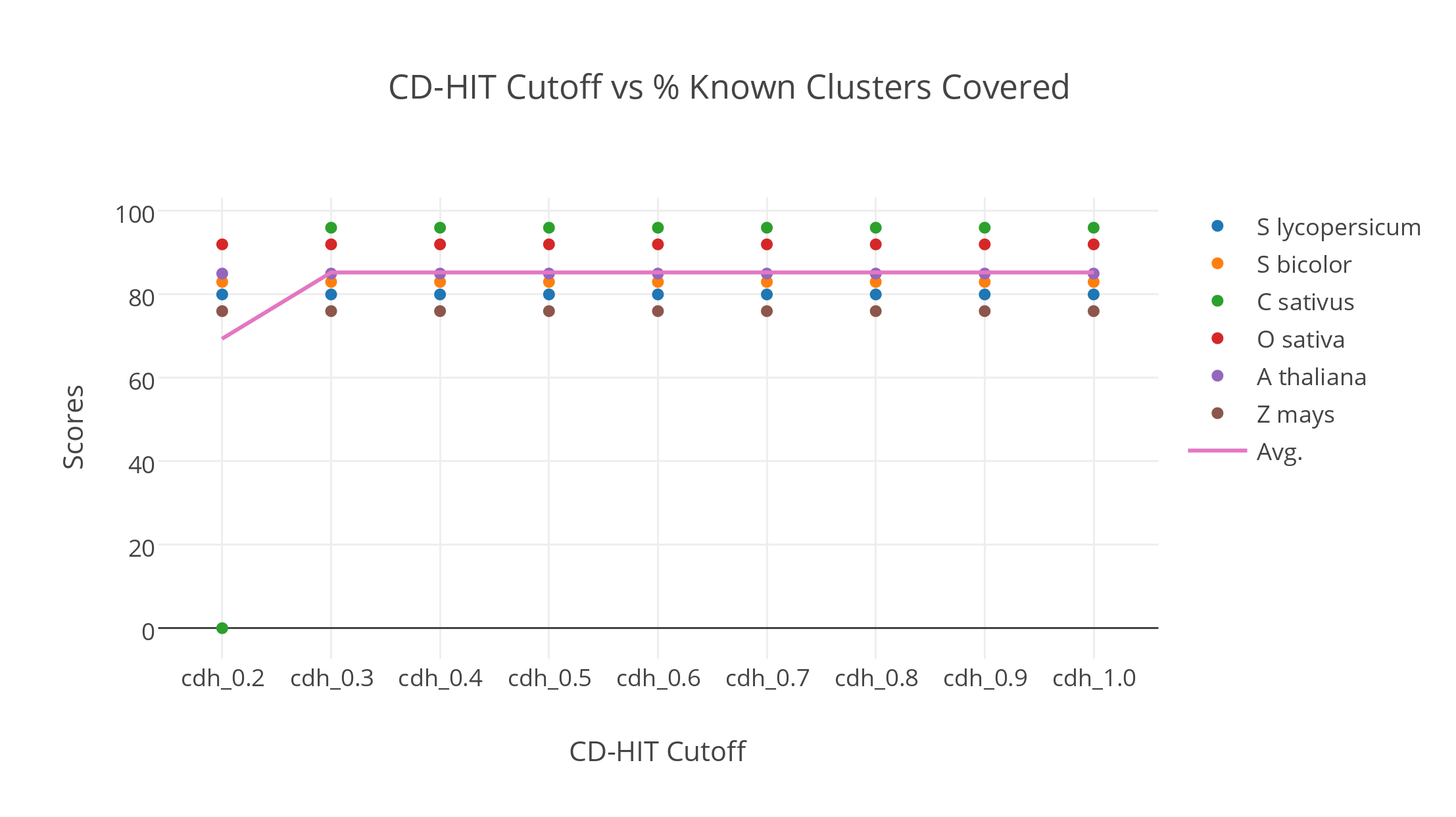


**SI Figure 1.** Effect of the CD-HIT cutoff parameter on the total number of predicted clusters and the coverage of known clusters. Coverage scores are calculated by comparing results of detected known clusters with literature (+1 for matching gene, -0.5 for absent gene, and -0.1 for extra gene; then converted to a percentage ratio). The default cut-off was chosen based on manual inspection of clusters that were gained/lost when changing the value of the parameter.


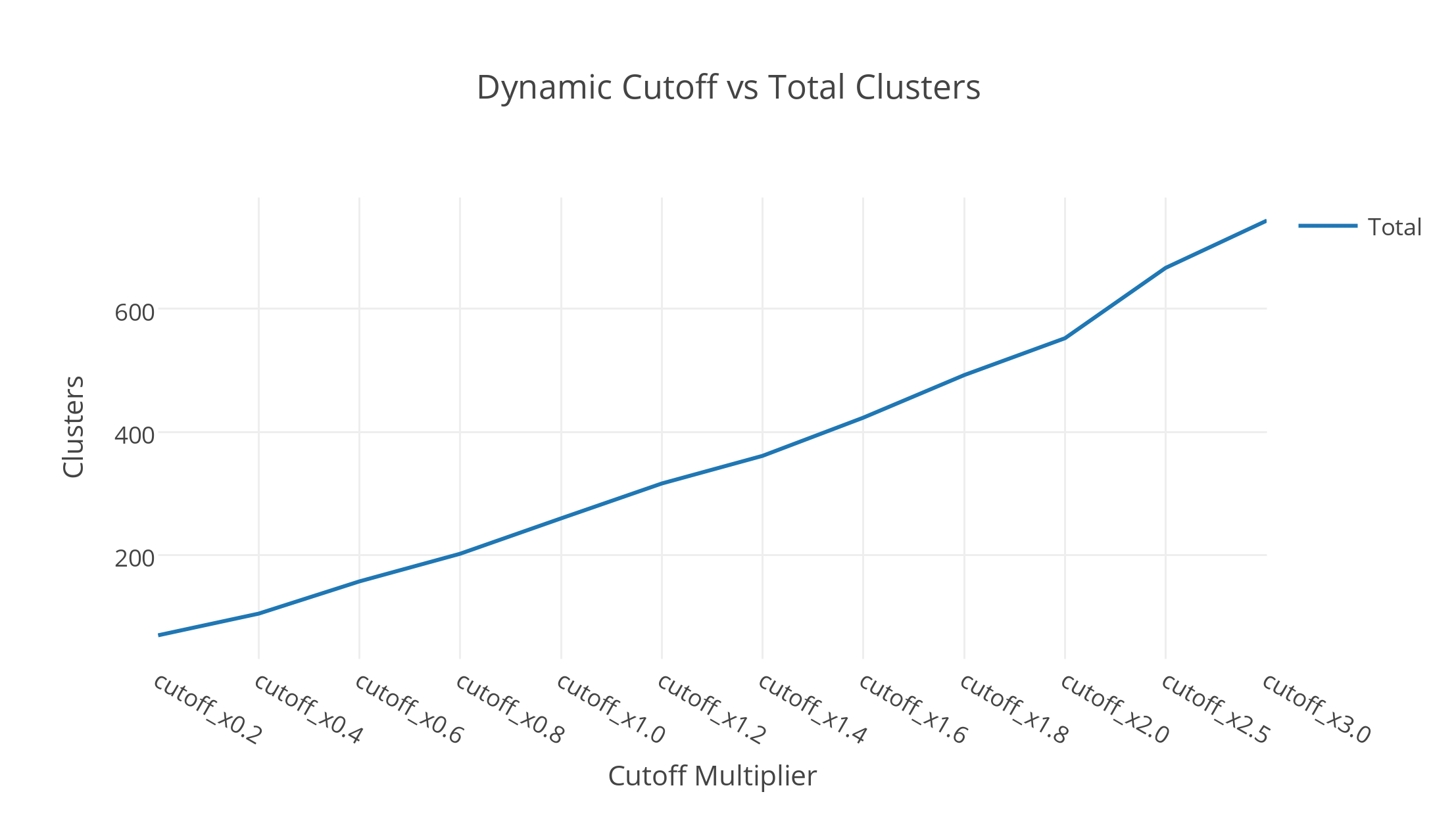

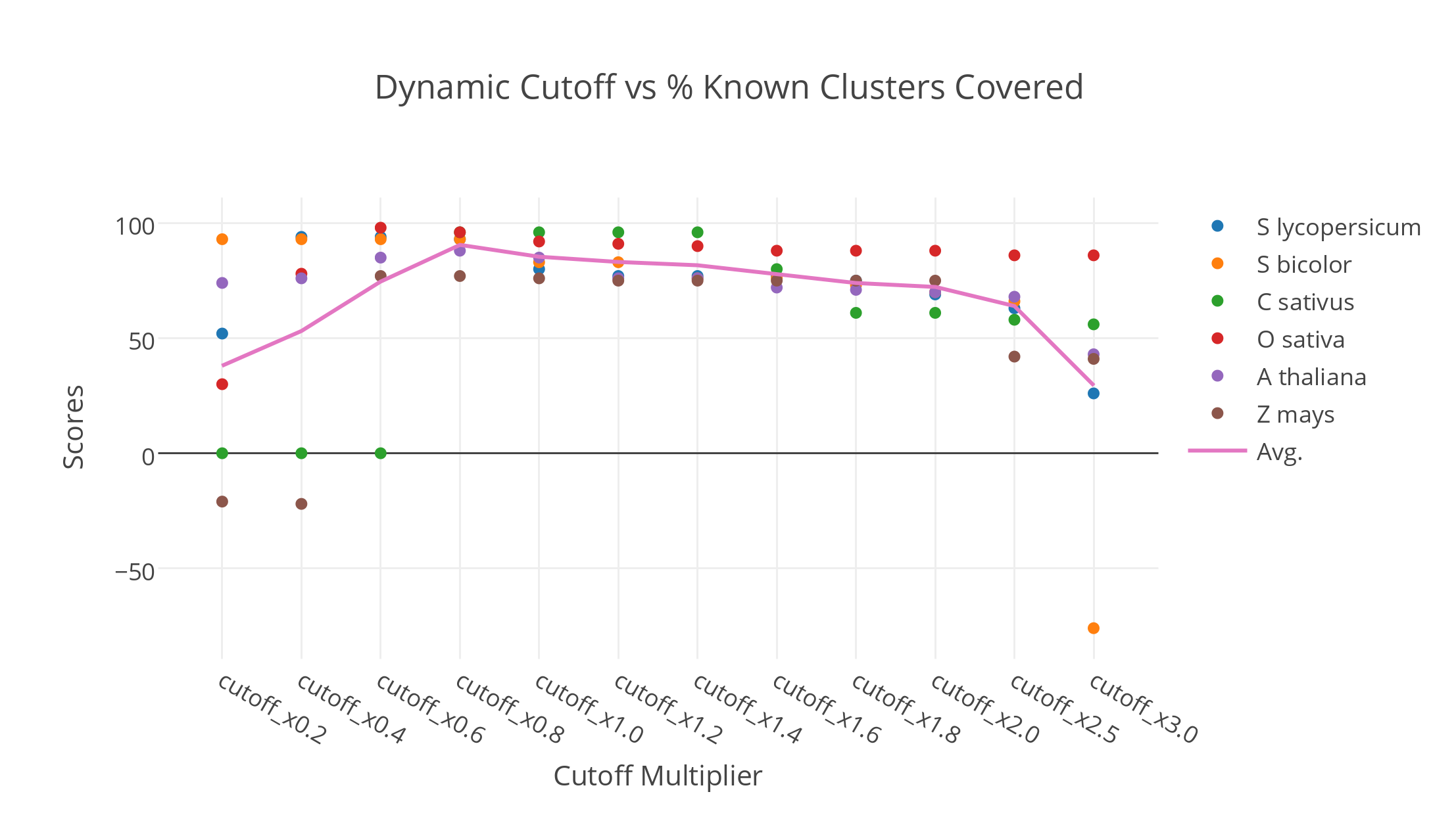


**SI Figure 2.** Effect of the dynamic cutoff parameter on the total number of predicted clusters and the coverage of known clusters. The default cut-off was chosen based on manual inspection of clusters that were gained/lost when changing the value of the parameter. Coverage scores are calculated by comparing results of detected known clusters with literature (+1 for matching gene, -0.5 for absent gene, and -0.1 for extra gene; then converted to a percentage ratio). The high score near the chosen default (lower panel) validates that the parameters effectively identify known gene clusters.

**
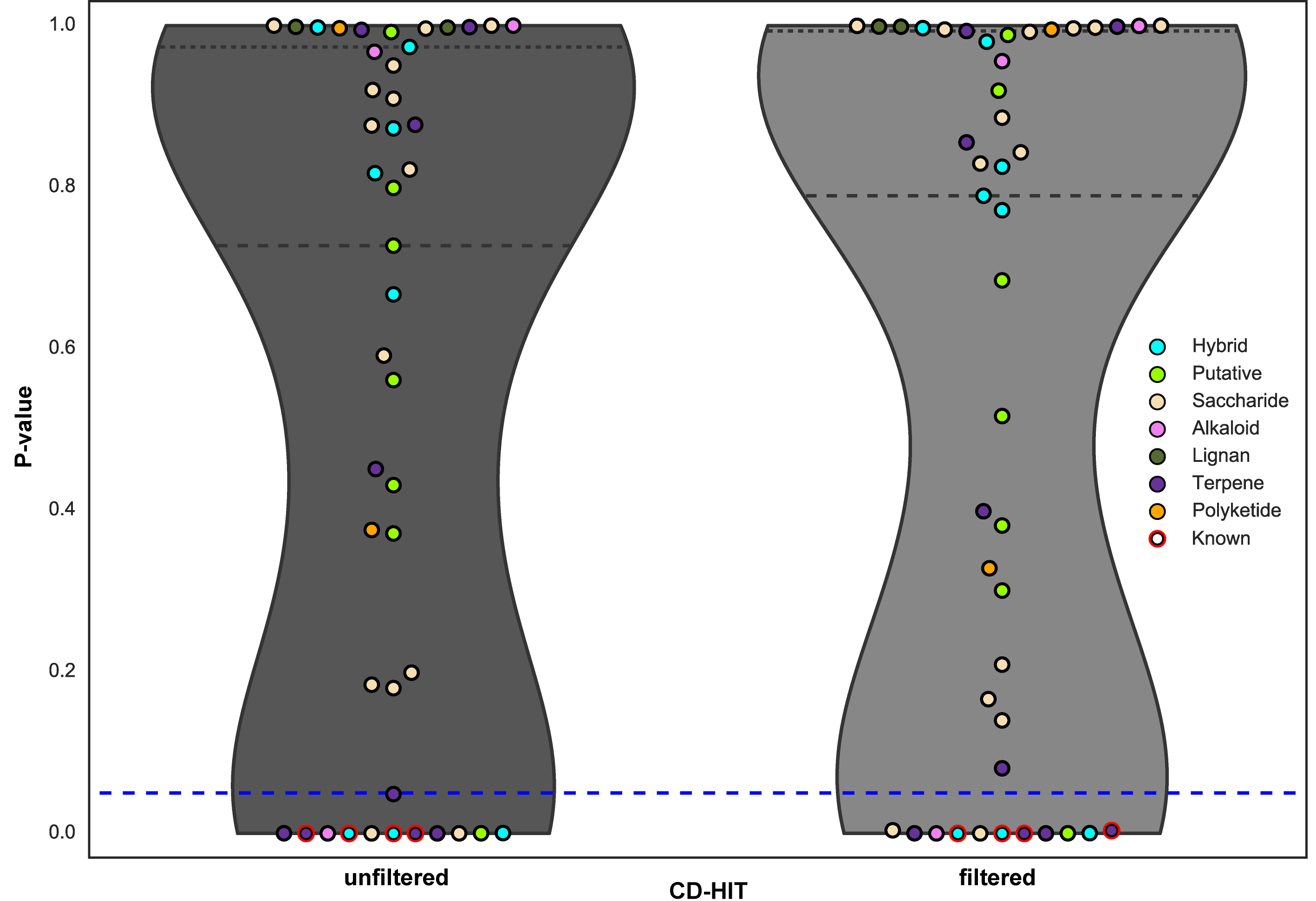
SI Figure 3.** Mann-Whitney U test results of the PCC distribution of every *A. thaliana* cluster against a background distribution. Each circle represents a cluster, with fill colors denoting its assigned cluster type. Circles outlined in red denote known clusters (Marneral, thalianol, tirucalla and arabidiol/baruol). The blue dashed line indicates the significance level (P=0.05), and the black dashed lines the second and third quartile of each P distribution. Left: P distribution without discarding gene-pairs in the same CD-HIT cluster. Right: P distribution when discarding gene-pairs in the same CD-HIT cluster. Multiple testing correction was consciously refrained from given the fact that each comparison was among different samples, with each background distribution tailored to the cluster size; still, even applying a strict Bonferroni correction would only lower the number of significantly coexpressed clusters from 11 to 10.
